# Widespread Compensatory Evolution Conserves DNA-Encoded Nucleosome Organization in Yeast

**DOI:** 10.1371/journal.pcbi.1001039

**Published:** 2010-12-23

**Authors:** Ephraim Kenigsberg, Amir Bar, Eran Segal, Amos Tanay

**Affiliations:** 1 Department of Computer Science and Applied Mathematics, Weizmann Institute of Science, Rehovot, Israel; 2 Department of Physics of Complex Systems, Weizmann Institute of Science, Rehovot, Israel; 3 Department of Molecular Cell Biology, Weizmann Institute of Science, Rehovot, Israel; McGill University, Canada

## Abstract

Evolution maintains organismal fitness by preserving genomic information. This is widely assumed to involve conservation of specific genomic loci among species. Many genomic encodings are now recognized to integrate small contributions from multiple genomic positions into quantitative dispersed codes, but the evolutionary dynamics of such codes are still poorly understood. Here we show that in yeast, sequences that quantitatively affect nucleosome occupancy evolve under compensatory dynamics that maintain heterogeneous levels of A+T content through spatially coupled A/T-losing and A/T-gaining substitutions. Evolutionary modeling combined with data on yeast polymorphisms supports the idea that these substitution dynamics are a consequence of weak selection. This shows that compensatory evolution, so far believed to affect specific groups of epistatically linked loci like paired RNA bases, is a widespread phenomenon in the yeast genome, affecting the majority of intergenic sequences in it. The model thus derived suggests that compensation is inevitable when evolution conserves quantitative and dispersed genomic functions.

## Author Summary

Purifying selection is a major force in conserving genomic features. It pushes deleterious mutations to extinction while conserving the specific DNA sequence. Here we show that a large proportion of the yeast genome evolves under compensatory dynamics that conserve genomic properties while modifying the genomic sequence. Such compensatory evolution conserves the local G+C content of the genome, which influences nucleosome organization. Since purifying selection is too weak to eliminate every weakly deleterious mutation in nucleosome bound or unbound sequences, the local G+C content is frequently stabilized by compensatory G+C gaining and G+C losing mutations in proximal loci. Theoretical analysis shows that compensatory evolution is inevitable when natural selection is weak and the genomic feature is distributed over many loci. These results imply that sequence conservation may not always be equated with overall selection. They demonstrate that cycles of weakly deleterious substitutions followed by positive selection for corrective mutations, which were so far studied mostly in RNA coding genes, are observed broadly and profoundly affect genome evolution.

## Introduction

With the complete sequencing of a large number of genomes, and with the rapid progress in the development and application of methodologies for functional annotation of whole genomes [1], it is becoming evident that our basic concepts of genomic function must be updated. The view of genomes as “bags of genes” is challenged by multiple lines of evidence, such as the extensive transcription of short and long RNAs from a substantial fraction of the genome [2]–[4], and the identification of a dense grid of enhancers and transcription factor binding sites in regions that could not be previously associated with genes [5], [6]. Some of the properties of the newly emerging genomic encodings are clearly different from the prototypic example of the triplet genetic code. The direct mapping between genomic positions (codons) and function (peptides) which is a hallmark of the genetic code does not seem to hold for the majority of the genome. Instead, genomic encodings integrate small contributions from multiple positions to form complex and quantitative outcomes. These types of *dispersed encodings* may be involved in defining enhancer sequences, maintaining epigenomic switches, affecting widespread transcription, and contributing to chromosome structure and dynamics. The evolutionary implications of these new types of codes are still poorly understood. The classical models in molecular evolution assume fitness to be a function of a single evolving locus. Conservation of the function encoded by such a locus is quantitatively predicted to decrease its rate of evolution. What rates of evolution can be expected when each of the multiple positions have small contributions to some joint quantitative fitness?

*Neutral compensatory substitutions* were predicted by Kimura 25 years ago [7] to couple substitutions in pairs of interacting protein coding loci. Kimura's concept was that an evolving population trajectory may visit suboptimal fitness levels transiently, thereby invoking an adaptive corrective force that can bring the system back to optimality. Such a process will change the genomic sequence, fixating pairs of compensatory alleles. Kimura's compensatory dynamic may work in any group of loci that are associated with an epistatic (non linear fitness function) constraint and was quantified extensively in RNA coding loci where the epistatic coupling of paired loci has a clear structural interpretation [8]–[10]. Another important source of genomic information, transcription factor binding sites, poses evolution with a different type of epistatic constraint by forming a quantitative binding energy landscape that affects gene regulation [11], [12]. The evolution of binding sites was shown to drive compensatory effects at the single site level [13] and also at the level of binding site clusters (or enhancers) [13], [14]. Studies of enhancer evolution are continuously providing striking examples for plasticity and compensation [15]–[17], but due to their heterogeneity, it is currently difficult to develop a general understanding of their evolutionary dynamics.

A simple experimentally characterized example of a dispersed genomic encoding involves the effect of DNA sequence on nucleosome organization [18], [19]. *In-vitro* and *in-vivo* experiments in yeast [20], [21] and other species [22]–[24] showed that nucleosomal packaging is correlated with preferential binding of nucleosomes to specific dinucleotide periodicities, and is strongly anti-correlated with A+T content in general and with poly(A/T) sequences in particular [20], [23], [25], [26]. The correlation between nucleosome occupancy and the underlying DNA sequence is sufficiently powerful to allow sequence based nucleosome occupancy prediction, but this prediction is not based on a strict requirement for certain nucleotides to appear at precise positions. Rather, information from multiple sequence positions along the 147bp length of the nucleosome contributes to the affinity of nucleosomes to a given sequence and consequently, to the formation of stable or semi-stable nucleosome configurations [27]. The evolution of these sequence determinants thus serves as a test case for the dynamics of dispersed genomic encodings. Analysis of substitution rates in yeast suggested that genomic sequences that are unbound to nucleosomes are evolving slower than genomic sequences that are bound to nucleosomes [20], [28]–[30]. Whether this is an indication of classical purifying selection on nucleosome encoding sequences, increased abundance of transcription factor (TF) binding sites at low nucleosome occupancy loci, or nucleosome-associated mutability, is currently unclear [31].

Here we analyze patterns of divergence and polymorphisms in yeast intergenic sequences to substantiate an extended model of selection on a dispersed genomic encoding. The analysis shows that yeast low nucleosome occupancy sequences have maintained a high A+T content throughout the evolution of the *Saccharomyces cerevisiae* lineage. Contrary to standard evolutionary models, we show that this conservation was made possible not by pointwise sequence conservation, but by a compensatory coupling of decreased rates of A/T-losing substitutions and increased rates of corrective A/T-gaining substitutions. Theoretical analysis suggests that this type of evolutionary dynamics is largely unavoidable when the genome employs dispersed functional encodings. The evolutionary dynamics we reveal shuffle sequences continuously while preserving their encoded function, creating a dynamic yet balanced process that may be central to the evolution of gene regulation.

## Results

### Regional heterogeneity in nucleotide composition is correlated with yeast nucleosome occupancy

**Figure 1:**
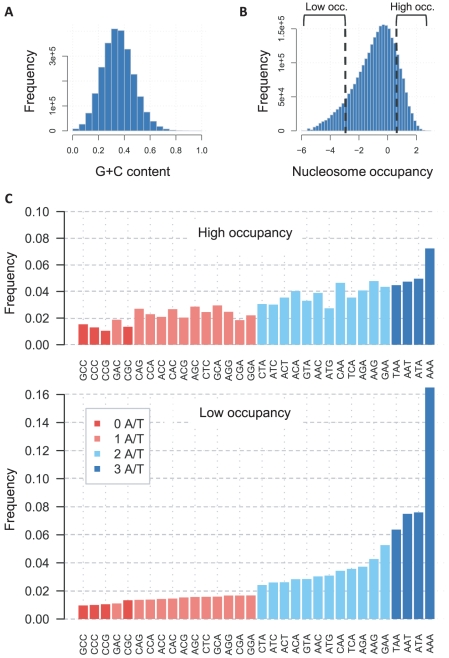
Yeast sequence heterogeneity is correlated with nucleosome occupancy. A) Heterogeneous local G+C content in yeast. Shown is the distribution of G+C content in small (20 bp) bins across intergenic sequences in the *S. cerevisae* genome (see **Fig S1** for further analysis). **B) Partitioning the genome into high and low occupancy sequences.** Shown is the distribution of *in-vivo* nucleosome occupancy scores across all yeast intergenic loci (data from Kaplan et al., 2009). **C) A/T trinucleotides are enriched at low occupancy loci.** The frequencies of all trinucleotides in loci with high (top) and low (bottom) nucleosome occupancy are depicted. As shown before, A/T trinucleotides are in excess at low occupancy loci. Also observed is the correlation between the number of G/C nucleotides within the trinucleotide and the relative abundance of the trinucleotide in high vs low occupancy loci.

The global G+C content of the yeast intergenic genome is about 35% (**Fig 1A**) but there is a significant heterogeneity in the genome local nucleotide composition (**Fig S1**). Such heterogeneity must be the consequence of a variable evolutionary process working in G+C poor and G+C rich sequences. Recently it was shown that nucleosome occupancy patterns strongly correlate with local G+C content in yeast [32]. We define *high nucleosome occupancy loci* as those in the top 21% MNase-seq coverage percentiles *in-vivo* (total 540 kbp, **Fig** **1B**), and *low nucleosome occupancy loci* as those in the bottom 14% MNase-seq coverage percentiles *in-vivo* (total 350 kbp). Overall, the intergenic G+C content at high occupancy sequences (∼40% G+C) is higher than the G+C content of low occupancy sequences (∼28% G+C). This heterogeneity is even more pronounced when studying the distribution of tri-nucleotides (**Fig 1C** **, Fig S2**), showing A/T tri-nucleotides to be more abundant in low occupancy sequences, and pointing towards additional nucleosome sequence preferences. It was shown before that *in-vitro* nucleosome occupancy can be robustly predicted from the distribution of 5-mers or even 3-mers in the sequence [21]. This suggests that the functionality and fitness contribution of DNA-encoded nucleosome organization, if such a contribution exists, is dispersed across multiple loci in a quantitative fashion and is not encoded by a strict requirement for precise sequence elements at one or a few positions. To prove or disprove the hypothesis that yeast intergenic G+C content heterogeneity is affected by nucleosome-related selection, we studied the evolutionary dynamics of yeast sequences bound and unbound to nucleosomes. We hypothesized that through characterization of these dynamics, we may reveal, in addition to the sequence constraints affecting yeast nucleosome organization, some general principles governing the evolution of dispersed genomic encodings.

### Analysis of context-dependent substitution rates reveals correlation between nucleosome occupancy and evolutionary dynamics

**Figure 2:**
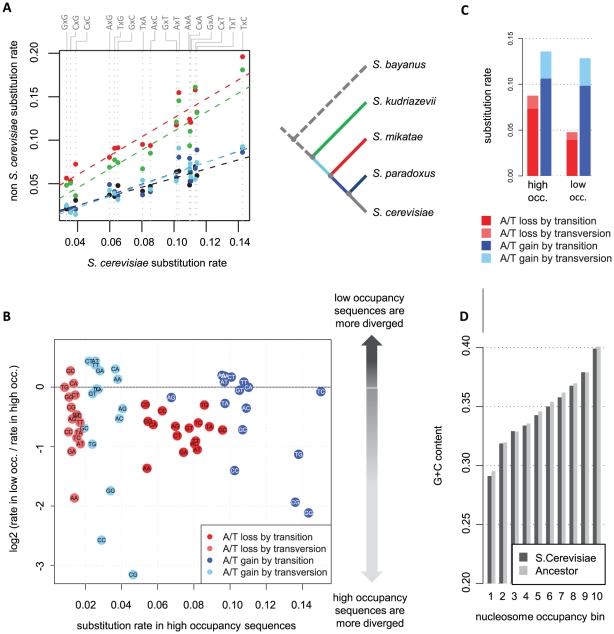
Low occupancy sequences lose A/T nucleotides slowly and gain them in a context-dependent fashion. A) Yeast substitution rates are robustly correlated with the flanking nucleotides. Shown are the inferred C to T substitution rates for the *S. cerevisiae* lineage (X-axis), and other *sensu stricto* lineages (color coded, Y-axis). Each point represents the C to T substitution rate in one of 4x4 different flanking nucleotide contexts which are defined using the 5′ and 3′ nucleotides depicted on top. The data reveal a four-fold variation in substitution rates at different contexts, which is consistent among the different lineages (as shown by the fit between the independently inferred substitution rates of *S. cerevisiae* and of the other lineages). Controlling for this variation is important when comparing substitution dynamics in A+T rich vs. A+T poor genomic regions, such as low and high occupancy sequences. **B) Different evolutionary dynamics in low and high occupancy loci.** Shown are log-ratios of substitution rates in low vs. high occupancy sequences (Y-axis) plotted against the substitution rates at high occupancy sequences (X-axis). Each point represents the rate of one of four types of substitutions (color coded) in loci flanked by the 5′ and 3′ nucleotide depicted inside the data point. Substitutions in reverse complementary contexts are averaged and shown only once. A/T-losing substitutions (red, pink) are ∼45% slower in low occupancy loci, an effect that is observed independently for transitions and transversions across the different flanking sequence contexts. A/T-gaining substitutions (blue, cyan) are highly dependent on the context, with the main group having rates which are independent of the nucleosome occupancy and with A/T gains in G/C flanking contexts highly conserved in low occupancy sequences. **C) Averaged substitution trends.** Shown are overall rates of A/T-gaining and A/T-losing substitutions in high and low nucleosome occupancy (occ.) averaged over all contexts. The simplified divergence pattern is difficult to explain using standard models of selection, since different types of substitution are differentially affected. **D) The S. cerevisiae lineage maintained the G+C content of low and high occupancy sequences.** Shown are the average G+C content in the extant *S. cerevisiae* genome and in the inferred common ancestor of *S. cerevisiae* and *S. paradoxus*, depicted for 10 levels of *S. cerevisiae* nucleosome occupancy (Methods). The analysis suggests that the highly variable substitution rates shown in B are not driving divergence in net G+C content but take part in a conservative process.

To study the evolutionary dynamics that underlie G+C content heterogeneity and nucleosome occupancy in the yeast genome, we inferred substitution rates and ancestral sequences in the *Saccharomyces sensu stricto* clade. We performed evolutionary inference from alignments of five yeast genomes [33], [34] for sequences that were classified as high nucleosome occupancy loci in *S. cerevisiae.* We separately inferred the evolutionary trajectory at low nucleosome occupancy loci. The analysis omitted exonic sequences, since the evolutionary dynamics in these involve additional sources of selection relative to those affecting intergenic sequences. Differences in locus mutability are known to be associated with the flanking nucleotides [35], [36], and this effect may severely bias the comparison of evolutionary dynamics between regions with different nucleotide composition. For example, A+T rich regions, like low-occupancy sequences, may exhibit slower divergence of A/T nucleotides than G+C rich regions, simply because A/T mutability is reduced in the flanking context of A/T nucleotides. To account for this effect of flanking nucleotides on substitution dynamics, we independently estimated the rate of substitution at all 16 possible combinations of flanking nucleotides. Indeed, the substitution rates estimated by our model vary significantly among flanking contexts both in high and low occupancy loci and reflect context-dependency that is consistent among phylogenetic lineages (**Fig 2A**, **Fig S3**). For example, the C to T transition rate over the *S. cerevisiae* lineage in low occupancy regions varies between ∼0.14 in the context of tCc and ∼0.03 in the gCg context. The estimation of context-dependent substitution rates proved essential for the unbiased comparison of evolutionary dynamics between the low occupancy, G+C poor, and the high occupancy, G+C rich sequences. As we show next, it allowed us to robustly identify and validate major differences in the evolutionary regimes of these two classes of loci.

**Figure 3:**
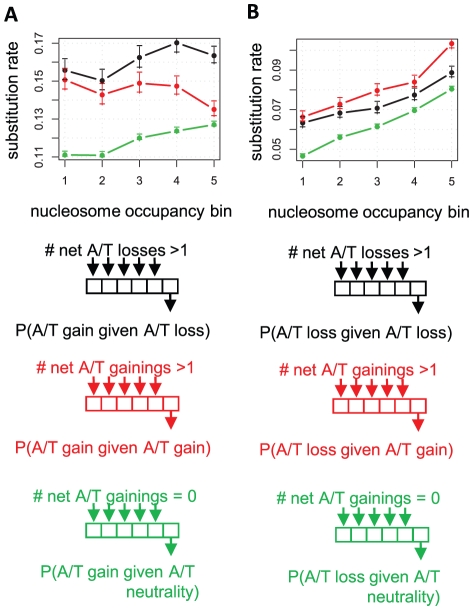
A/T-gaining and A/T-losing substitutions are spatially coupled. A) A/T gain rates are faster next to inferred A/T loss events. Shown is a comparison of the rate of A/T gaining substitutions near inferred sites of A/T-losing (black) and A/T-gaining (red) substitution (Methods), plotted for different ranges of nucleosome occupancy (X-axis). The rate of A/T gain near conserved loci is shown for reference (green). We observe an elevated rate of A/T gain near A/T-losing sites. **B) A/T loss rates are faster next to A/T gain events.** Similar analysis of A/T losing substitution rates around inferred A/T gain and A/T loss events.

### Low occupancy sequences lose A/T nucleotides slowly but gain A/T nucleotides at the same rate as high occupancy sequences

We first studied *S. cerevisiae* substitution rates inferred from intergenic sequences within 200 bp of annotated transcription start sites. It is known that this region in yeast promoters is enriched for transcription factor binding sites and exhibits a stereotyped nucleosome-depleted region of length ∼100–150 bp. As shown in **Fig 2B–C** (see also **Fig S4 and Fig S5**), the analysis reveals that the rates of A/T-losing transitions (A to G, T to C) and transversions (A to C, T to G) are ∼45% lower in low occupancy sequences than in high occupancy sequences. A decrease is observed for all 16 nucleotide contexts (within an estimation variance), and is slightly more pronounced in A/T contexts (AAA, AAT). Notably, the rates of A/T-gaining transitions (G to A, C to T) and transversions (G to T, C to A) are not decreased like the A/T-losing substitutions. In most sequence contexts, the rates of A/T-gaining substitutions are higher in low occupancy sequences or similar between the sequence classes. On the other hand, when flanked by G's or C's, the rates of A/T-gaining substitutions are four times slower in low occupancy compared to high occupancy sequences. Evolutionary theory could not predict these dynamics if the evolution of G+C content was neutral (unless an extremely unlikely mutational regime is separating high from low occupancy regions, as we disprove below using population genetics data). Moreover, a simple theory assuming average stronger evolutionary constraint on low occupancy sequences [20], [29] would predict a general decrease in the substitution rates in the region and would not explain the asymmetry between A/T-gaining and A/T-losing substitution rates.

### Overall G+C content is conserved for high and low nucleosome occupancy DNA

An important assumption underlying our evolutionary analysis above is that the evolutionary regime operating in regions that are occupied (or unoccupied) by nucleosomes in the extant *S. cerevisiae* genome has been the same since the divergence of *S. cerevisiae* from *S. paradoxus*. Violations of this assumption can potentially affect our substitution rate estimations. For example, if nucleosome occupancy is determined by the genomic sequence, but is not under selection, nucleosomes may drift freely following substitutions spontaneously generating new A+T rich hotspots. Following that, we may enrich for substitutions that increase A+T content in extant low occupancy sequences by assuming nucleosome organization were conserved. To verify that such a scenario has not significantly affected our analysis of TSS-proximal substitution rates, we inferred the G+C content in the common ancestor of *S. cerevisiae* and *S. paradoxus*, for 10 ranges of *S. cerevisiae* nucleosome occupancy levels, and compared it to the extant G+C content (**Fig 2D**). We found that the G+C content at all levels of nucleosome occupancy did not change significantly during evolution in the *S. cerevisiae* lineage. Sequences proximal to TSSs therefore conserve their regional G+C content (at least on average). Consequently, the different rates of substitutions in high and low nucleosome occupancy loci do not represent net divergence in the sequence features that correlates with nucleosome occupancy. This is further confirmed by recent comparative analysis of nucleosome organization in *S. cerevisiae* and *S. paradoxus*, which revealed only limited divergence in nucleosome positioning for these species [37], [38]. The highly non symmetric substitution dynamics observed at different levels of nucleosome occupancy must therefore be explained by means of a stationary evolutionary process that conserves the underlying nucleosome-associated encoding.

### Spatial coupling between A/T-losing and A/T-gaining substitutions suggests compensatory evolution preserves high and low occupancy sequences

One intriguing possibility that may explain the asymmetry between the rates of A/T-losing and A/T-gaining substitutions in low occupancy sequences is that while A/T-losing mutations are selected against, some can be sustained in the population. Consequently, positive selection is able to push to fixation corrective A/T-gaining mutations (possibly at different genomic positions). If this hypothesis is correct, we can predict that loci near sites of A/T-losing substitutions will be enriched with A/T-gaining substitutions and vice versa. Remarkably, the yeast divergence patterns confirm this prediction. The data reveal that rates of A/T-gaining substitution are accelerated next to sites of observed A/T loss (compared to rates near conserved loci, **Fig 3A**). Furthermore, as shown in **Fig 3A**, this effect does not represent general spatial coupling of substitutions, since the A/T gain rate is significantly higher near sites of A/T loss than it is near sites of A/T gain. Conversely, the rates of A/T losing substitutions are higher next to sites of observed A/T gain (**Fig 3B**). Unexpectedly, this coupling effect is observed robustly across the entire spectrum of nucleosome occupancy levels (p<1e-5 for high nucleosome occupancy, p<0.04 for low nucleosome occupancy). The coupling between contrasting substitutions on spatially linked loci suggests the involvement of a common selective constraint, without which the dynamics at these loci must be independent of each other. The data therefore suggest that compensating A/T-losing and A/T-gaining mutations work to conserve a heterogeneous G+C content (both high and low) in TSS-proximal sequences.

### Compensation and possible divergence of low occupancy regions revealed by the substitution dynamics at TSS-distal sequences

**Figure 4:**
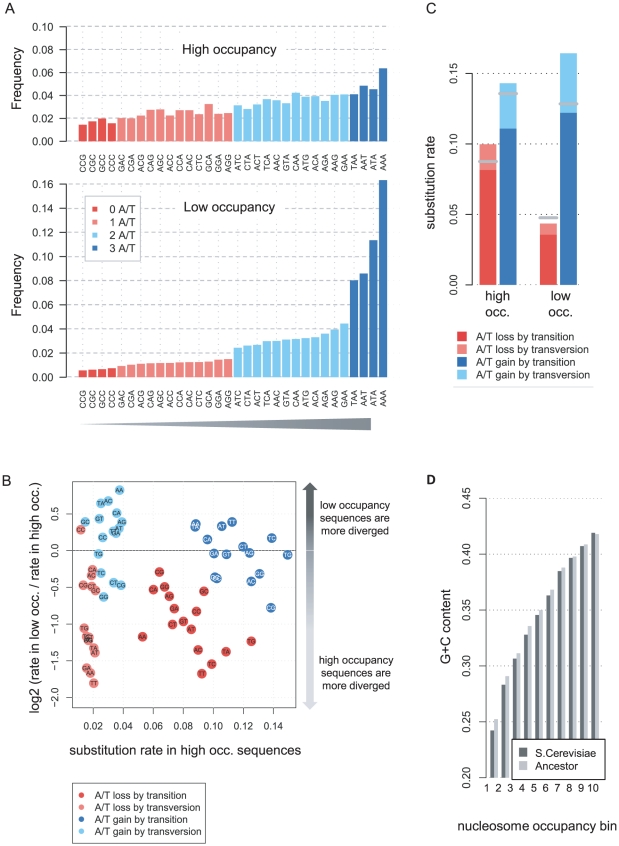
Compensatory evolution at TSS-distal sequences. **A**) **The TSS-distal trinucleotide spectrum is modified.** Shown are trinucleotide frequencies at TSS-distal high and low occupancy sequences. Compared to the distribution at TSS-proximal sequences, the low occupancy sequences contain more A/T trinucleotides and less G/C trinucleotides. **B**) **TSS-distal low occupancy sequences lose A/T slowly and gain them rapidly.** Shown are ratios of substitution rates in low vs. high occupancy sequences (Y-axis) plotted against the substitution rates at high occupancy sequences (X-axis). Each point represents the rate of one of four types of substitution (color coded) in loci flanked by the 5′ and 3′ nucleotide depicted inside the data point. A/T losing substitutions (red, pink) are consistently slower in TSS-distal low occupancy loci, with very similar dynamics to those observed in TSS-proximal sequences (compare **Fig 2**). A/T gaining substitutions (blue, cyan) generally occur more rapidly in low occupancy loci than in high occupancy loci. A/T gains in G/C flanking contexts are somewhat conserved, though not to the extent observed in TSS-proximal low occupancy loci. **C**) **Averaged substitution rates.** Shown are the rates of A/T-gaining and A/T-losing substitutions at TSS distal (bars) and TSS proximal (gray ticks) high and low occupancy sequences, averaged over all flanking contexts. **D**) **Evolution of G+C content at different occupancy levels.** Shown are average G+C contents in the extant *S. cerevisiae* genome and in the inferred common ancestor of *S. cerevisiae* and *S. paradoxus*, depicted for 10 levels of nucleosome occupancy (Methods). An overall conservation of G+C content is observed. Conservation is disrupted for low occupancy sequences, suggesting that some of the low occupancy TSS-distal sequences in *S. cerevisae* have decreased their G+C content recently.

The trinucleotide distributions of low occupancy TSS-distal sequences (over 200 bp from an annotated TSS) are generally similar to those in TSS-proximal loci, but some important differences are notable (**Fig 4A**). First, for low occupancy sequences, G/C trinucleotides are rarer in TSS-distal than in TSS-proximal loci. Second, poly-A/T trinucleotides are enriched relative to other A/T rich nucleotides in TSS-proximal but not TSS-distal low occupancy loci. These differences may represent a lower fraction of TF binding sites in TSS-distal regions [19], [20] (**Fig S6** for additional analysis). As shown in **Fig 4B–C**, TSS-distal A/T-losing substitution rates are decreased in low occupancy vs. high occupancy sequences, consistent with the observations in TSS-proximal loci. Furthermore, the rates of A/T-gaining substitution in many contexts are increased in low occupancy vs. high occupancy sequences, similar to their behavior in TSS-proximal regions (but with G/C-flanking contexts not highly conserved). Comparison of the ancestral and extant G+C content reveals conservation at high levels of nucleosome occupancy, but some average decrease in G+C content for low nucleosome occupancy loci (**Fig 4D**). Analysis of compensatory spatial correlation between A/T-gaining and A/T losing substitutions reveals significant coupling at high nucleosome occupancy levels (p<6e-4). Also shown is the tendency of A/T-gaining substitutions at low nucleosome occupancy to occur in clusters (**Fig S7**).

The data therefore support a compensatory substitution process that drives G+C content conservation in most TSS-distal loci, in a way analogous to the dynamics at TSS-proximal loci. This is demonstrated by the asymmetric rates of A/T gain and A/T loss, the conservation of G/C content and the compensatory substitution coupling at most ranges of nucleosome occupancy. An exception to this general trend is observed at some of the TSS-distal low occupancy loci. We hypothesize that during the evolution of the *S. cerevisiae* lineage, de-novo A/T-rich hotspots may have driven divergence of nucleosome organization in some TSS-distal loci (possibly since these were under weaker selection [37], [38]). This effect may explain the non-stationary G+C content and spatial clustering of A/T-gaining substitutions at extant TSS-distal low occupancy loci (**Fig S7**). Taken together, the data on TSS-distal sequences further support the idea that selection maintains heterogeneous G+C content across most yeast intergenic sequences (and in particular at TSS-proximal sequences), and that this selection drives changes in substitution rates that are difficult to explain using models of selection on a single locus.

### A theoretical model recapitulates the empirical yeast evolutionary dynamics

**Figure 5:**
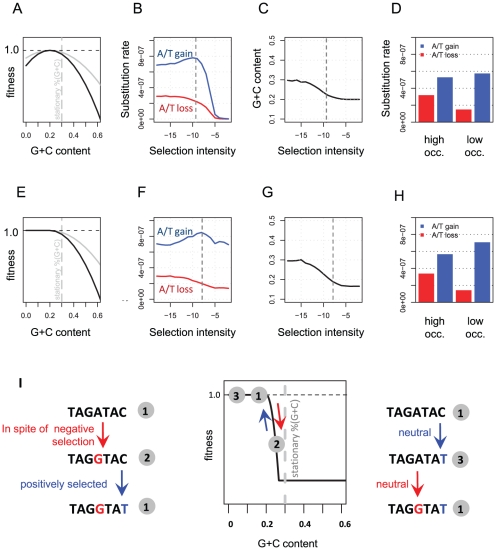
A model of weak compensatory selection predicts the evolutionary dynamics at yeast low occupancy sequences. We simulated the evolution of a fixed size population of small (20 bp) “genomes” in simple fitness landscapes that depend only on the G+C content of the sequence (Methods). We used a mutational input that favors A/T over G/C, resulting in a neutral stationary G+C content of 30%. **A**) **G+C goal fitness landscape.** Shown are fitness landscapes (fitness value as a function of the G+C content) preferring low G+C content (the “low occupancy” regime). We generated regimes with different selection intensities (denoted by η) by changing the slope of the depicted parabola (shown are the landscapes for two different intensities). **B**) **Substitution rates.** Shown are substitution rates of A/T loss (red) and A/T gain (blue) measured in populations evolving under different levels of selection intensities (X axis) in the low G+C content fitness landscape. We note the increase in A/T gain rate to values higher than neutral at intermediate selection intensities. **C**) **Stationary G+C content.** Shown are the population average G+C contents for population evolving in different selection intensities (X-axis), showing that at intermediate levels of selection where A/T gain rate were elevated (dashed line), the average G/C content is only slightly higher than the optimum. **D**) **Substitution rates for intermediate selection intensity.** We summarize the simulation by showing substitution rates for a specific level of selection intensity (marked in dashed lines in B and C). Data is shown for the low G+C fitness landscape and for a high G+C fitness landscape that was defined symmetrically with a preferred G+C content of 40% (**Fig S8**). The data are generally compatible with our empirical observations on yeast divergence rates (**Fig 2C** **,** **4C**). **E–H**) **Evolutionary dynamics for a threshold fitness landscape.** An analysis similar to the above, but with a threshold-function fitness landscape (E) reveals that an increase in A/T gaining substitution rates can be observed over a wide range of fitness intensities. **I**) **Compensatory evolution explains the increase in A/T gaining substitution rates.** According to our model, the evolution of low occupancy sequences is attempting to maintain low G+C content in spite of a flux of slightly deleterious mutations that pushes toward a higher stationary G+C content. Selection may not be sufficiently powerful to purge every deviation from the optimum and mutations that decrease A/T content may persist (even partially) in the population. These mutations trigger adaptive evolution of corrective mutations, which is efficient since it can occur at multiple positions. The schematic shown here assumes evolution in the threshold fitness landscape (E–H), in which A/T gaining substitutions are never deleterious and therefore robustly increased in rate even if selection is intensive. A variation of the same argument shows why A/T gain rate increases in the fitness landscape of A–D.

To study the hypothesis that selection on dispersed nucleosome encodings drives asymmetric substitution patterns in yeasts, we devised a simple theoretical model (**Fig 5**). We assume that a population of 20 bp sequences (each representing a different “genome”) is evolving given a constant flux of mutations in some fitness landscape that depends only on the G+C content of the sequence. The mutations transform G/C nucleotides to A/T nucleotides faster than they transform A/Ts to G/Cs, driving the genomes' stationary G+C content to a neutral level of 30%. Working against this stationary G+C content, the fitness landscape defines a lower G+C content (20%) as optimal, with symmetrically decreasing fitness for suboptimal values. This landscape is designed to approximate the potential selective pressure on low nucleosome occupancy sequences. We studied the model behavior at various selection intensities both analytically and using computer simulations (Methods). For each intensity level, we determined the A/T gain and A/T loss substitution rates and stationary G/C content (**Fig 5A–D**). When selection is weak, the dynamics we observed are neutral, with the rates of substitutions being equal to the rates of mutations, and the G+C content converging to the neutral stationary G+C content (30%). In contrast, when selection is strong, the rates of both A/T gain and A/T loss decrease to zero and the G+C content is optimal (20%). These two regimes are compatible with the standard evolutionary theory of selection on a single locus. More notable are the substitution rates observed at intermediate levels of selection. When selection is not sufficiently strong to purify all A/T-losing mutations, A/T-losing substitution rates are only partially decreased. Interestingly, this decrease is matched by an *increase* in the rate of A/T-gaining substitutions to levels higher than the neutral rate. The new balance between A/T-losing and A/T-gaining rates is sufficient to stabilize the G+C content of the regime at near-optimal levels. Detailed analysis reveals that the increase in the rate of A/T-gaining substitutions is driven by cycles of A/T-loss mutation at one position, which are corrected by an A/T-gain mutation at another position. Similar but opposite dynamics are observed when the optimal G/C content is higher than the neutral one (modeling selection of high G+C content in high nucleosome occupancy sequences, **Fig S8**). Furthermore, the compensatory regime is observed over a much wider range of selection intensities when the fitness landscape is more tolerant as shown, for example, in **Fig 5E–I**. These theoretical predictions are consistent with the empirical behavior observed in yeast, showing that weak selection can be sufficiently powerful to increase specific substitution rates over the neutral level due to a compensatory regime.

### Compensatory dynamics are supported by *S. cerevisiae* polymorphism data

**Figure 6:**
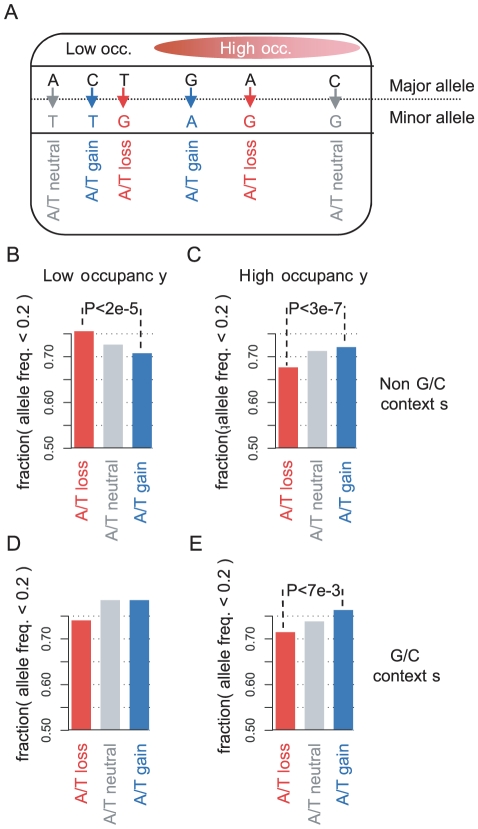
SNP data support the compensatory evolution hypothesis. Data [39] on allele frequencies in a sample of *S. cerevisiae* strains was used to test the hypotheses that low and high occupancy sequences maintain their local G+C content due to weak selection. Theory predicts that allele frequencies of deleterious mutations would tend to be smaller than frequencies of neutral mutations and that SNPs representing beneficial mutations would be frequently observed at higher allele frequencies. **A**) **Classifying SNPs.** Major and minor alleles at SNPs representing postulated A/T gain and A/T loss at low and high occupancy loci were determined as illustrated. Loci with G/C flanking context were analyzed separately. **B–E**) **Allele frequencies.** The groups of SNPs were compared by computing the fraction of SNPs with minor allele frequency smaller than 20%. Shown is the fraction of rare alleles in cases of A/T gain, A/T loss and A/T neutral polymorphisms in non G/C contexts in low occupancy sequences (B), non G/C contexts in high occupancy sequences (C), G/C contexts in low occupancy sequences (D), G/C contexts in high occupancy sequences (E). The data show A/T losing SNPs tend to be rarer than neutral SNPs and A/T gaining SNPs in low occupancy sequences. The opposite behavior is observed at high occupancy sequences or at G/C contexts, confirming the predictions of our evolutionary model.

Our evolutionary analysis above supports the idea that high and low nucleosome occupancy sequences in yeast evolve under a selective pressure to maintain their G+C content, or a refined nucleosome sequence potential that is approximated by the average G+C content. According to this scenario, in low occupancy sequences, which are generally A+T-rich, A/T-losing substitutions are weakly selected against, while A/T-gaining substitutions are frequently pushed to fixation by an adaptive force. According to our simulations and to the standard population genetics theory, such selection on A/T-gaining and A/T-losing mutations should affect the distribution of allele frequencies in the population. In low occupancy loci, A/T-losing single nucleotide polymorphisms (SNPs) are expected to have lower allele frequencies than A/T-neutral SNPs, while A/T-gaining SNPs should have higher allele frequencies. Analysis of polymorphic sites in a sample of 39 *S. cerevisae* strains [39] confirmed these predictions (**Fig 6**). We used data on 9185 SNPs in low occupancy loci and 16956 SNPs in high occupancy loci, approximating the minor allele frequency using majority voting and discarding sites with incomplete data or more than two alleles. In low occupancy loci, A/T-losing SNPs are more rare (<20%, alternative threshold generated similar results, **Fig S9**) than A/T-gaining SNPs in non G/C flanking context (p<2e–05). A reciprocal effect is observed at high occupancy loci, where A/T-gaining SNPs are more rare than A/T-losing SNPs in non G/C flanking context (p<3e–07). The reciprocality of the effect also confirms that our conclusions are not affected by general biases in the estimation of allele frequencies due to systematic sequencing errors. We note that as expected by the low divergence of A/T nucleotides in G/C flanking contexts of low occupancy sequences, the allele frequencies of A/T-gaining SNPs in such loci are reflective of stronger selection. This may be related to the enrichment of such flanking contexts at TF binding sites, as we discuss below.

## Discussion

### The evolutionary origins of G+C content heterogeneity in yeast intergenic regions

We classified yeast intergenic regions according to their nucleosome occupancy, and used evolutionary analysis of context-dependent substitution rates to reveal remarkable variability in the evolutionary dynamics of sequences bound and unbound to nucleosomes. Our analysis shows that low occupancy sequences lose A/T nucleotides slowly compared to high occupancy sequences, but gain A/T nucleotides at similar rates. We also observe spatial coupling between substitutions that gain A/Ts and substitutions that lose them, which suggests that a compensatory process preserves G+C content at both high and low occupancy loci. These observations are compatible with a model in which the local G+C content in yeast is conserved through weak quantitative selection. Such weak selection allows occasional fixation of substitutions that disrupt the optimal G+C content of the region, but then respond by adaptive evolution of corrective mutations at the mutated locus or at any of the surrounding genomic positions. Data on allele frequencies of yeast SNPs independently confirm the predictions of such a model. This set of observations proves that the G+C heterogeneity of yeast intergenic sequences is not a consequence of a neutral process and suggests that nucleosome organization may play a major role in this lack of neutrality.

### Selection and the signals for nucleosome organization

The role of DNA encoded nucleosome occupancy in regulating gene expression is difficult to isolate experimentally, mostly due to the challenge of separating cause and effect inside the complex system involving nucleosomes, remodeling factors and TFs. Previous analysis identified an anti-correlation between nucleosome occupancy and genomic conservation in yeast [20], [28]–[30] putting forward the hypothesis that low occupancy regions (nucleosome free regions, linkers) may be under selection, either due to their increased frequency of TF binding sites, or since they serve as anchors that organize the entire nucleosome landscape. According to our analysis nucleosome occupancy is tightly correlated with substitution patterns reminiscent of selection throughout the genome and not just at low occupancy regions. The data therefore strongly support the non-negligible contribution of DNA encoded nucleosome organization to fitness and therefore to genome regulation. This is further demonstrated by contrasting the G+C content related selection patterns at TSS-proximal sequences (**Fig 2** **,** **3**), with the frequent cases of overall divergence of A/T rich hotspots and clustered A/T-gaining substitution in TSS-distal low occupancy sequences (**Fig 4**). The data suggest that when selection is not working, nucleosome occupancy drifts following changes in the encoding sequences [37], [38]. We note that according to our simulations and the empirical data, the selection on nucleosomal sequences must be weak, driven by the very small (but still specific) fitness contribution of any individual genomic position. We predict that such selection is sufficiently powerful to contribute significantly to the heterogeneity of the yeast intergenic sequences, but it is clearly much weaker (per base) than the selection working to conserve classical functional elements. These theoretical considerations underline the difficulty in proving the functionality of specific nucleosome positioning sequences using direct genetics experiments, which typically require large and easily quantifiable phenotypic effects for specific genetic manipulations.

### Combined selection on TF binding sites and nucleosome positioning sequences in TSS-proximal low occupancy sequences

One source of evolutionary constraint on yeast intergenic sequences is their interaction with transcription factors. TF binding sites are known to be conserved among yeast species [33], [34] and their increased concentration in TSS-proximal nucleosome free regions was previously proposed to impose overall conservation at these regions. According to our inferred evolutionary dynamics at TSS-proximal DNA, selection on TF binding sites indeed contributes to the evolution of low occupancy sequences. This is indicated, for example, by a very low A/T gain rates in G/C trinucleotides (**Fig 2**), which are part of some of the most abundant and conserved yeast binding sites (e.g., Ume6, PAC, Reb1, MBP1) [11], [12], [40]. Nevertheless, selection on binding sites, even those that are A/T rich (e.g. TATA boxes) is highly unlikely to explain the nucleosome occupancy-dependent substitution rates we observed throughout the yeast genome. Specifically, the compensatory coupling of A/T-losing and A/T-gaining substitutions is not compatible with any particular binding site model. We therefore hypothesize that a combination of purifying selection on TF binding sites (either strong [33], [34] or weak [11]) and composite selection on DNA encoded nucleosome organization together define a complex fitness landscape that shapes the evolution of yeast intergenic sequences.

### Evolution of dispersed sequence encodings necessitates compensatory dynamics

We studied here a model of evolution as manipulating sequences in a complex fitness landscape that combines contributions from multiple coupled loci into a single *dispersed encoding*. As shown by theoretical and empirical analysis of the model, when selection on each individual locus is weak, purifying selection is incapable of completely purging mutations that are only slightly deleterious and these are continuously challenging the overall optimality of the sequence. This suboptimality is compensated effectively by adaptive evolution at multiple other loci that participate in the dispersed encoding. In contrast to other cases of compensatory evolution (proteins [41] or RNA molecules [8]-[10], [42]), the encodings we studied here provide ample direct ways to correct a slightly deleterious substitution, thereby increasing the rate of compensation. Our study builds on earlier work on codon bias [43], [44], but uses the global and experimentally characterized sequence classes at high and low nucleosomes occupancy loci to establish compensatory evolution as a major driving force in evolution under multi-site selection. This type of evolutionary dynamics may be generalized to other dispersed functional encodings [45], [46] including complex regulatory switches that typically involve a large number of TF binding sites of variable factors and specificities. The remarkably global nature of the compensatory effect we observed in yeast, which cause a measurable global increase in the substitution rate of specific mutations, supports the notion of an evolutionary process that conserves function without a strict requirement to conserve sequence. It is tempting to speculate that such a process may allow genomes to maintain diversity and continuously search the sequence space, without significantly compromising their existing regulatory circuits. Furthermore, this process may reduce, through compensation, the mutational load [47] resulting from the use of multiple loci to encode regulatory functions.

## Methods

### Data sets

Multiple alignments of the *Saccharomyces cerevisiae, Saccharomyces paradoxus*, *Saccharomyces mikatae*, *Saccharomyces kudriavzevii* and *Saccharomyces bayanus* were downloaded from the UCSC database [48] (sacCer2 version). Alignments were based on the SGD June 2008 assembly. A genome wide *in-vivo* nucleosome occupancy profile for *S. cerevisiae* was used as previously described [21], indicating a nucleosome occupancy value for each genomic position. SNP data were downloaded from the SGRP website [39]. Gene Annotations and transcription start sites of *S. cerevisiae* were taken from the SGD known gene table which corresponds to sacCer2 [49]. Transcription factor binding sites were downloaded from the UCSC Genome Browser [48] and are based on the chip-chip experiments described before [50].

### Classifying low and high occupancy sequences

Our analysis focused on intergenic genome sequences which are defined based on the SGD gene annotations. Each intergenic locus was defined as *TSS-proximal* if it is not part of an exon, and has an annotated TSS within 200 bp of it. TSS-distal loci included the remaining non exonic loci. We defined *low occupancy* loci as positions with nucleosome occupancy value lower than −2.5 (relative to the genomic mean, detailed description in Kaplan et al. [21]) and *high occupancy* loci as positions with occupancy higher than 0.4. Alternatively, we classified all loci to equal sized bins of nucleosome occupancy (ten in analysis of ancestral G+C context and five in the analysis of spatial coupling). Alternative definition of low occupancy linker regions based on raw data of MNase restriction sites resulted in similar results (data not shown).

### Estimation of substitution rates

As described in the text, a refined context dependent substitution model is essential for the correct estimation of the different evolutionary dynamics in low G+C content, low occupancy loci and high G+C content, high occupancy loci. We therefore applied a flexible substitution model to perform ancestral inference and learn evolutionary parameters from alignment data (details available upon request). The model included parameters for the substitution rates at each of 16 possible contexts parameterized by the identities of the 3′ and 5′ flanking nucleotides. Independent substitution rates were assumed for each lineage in a phylogenetic tree which was fixed throughout the process. We note that the model does not assume parametric constraints on different substitution rates, and infers substitution rates on lineages, rather than a global substitution rate matrix and branch lengths. This approach has proved more robust given that a sufficient number of loci was available to learn robustly the parameters at each lineage, and given that the substitution process in the different lineages indicated gradual changes in dynamics that a model using a universal rate matrix could not have accounted for (for example, the extant G+C content in each of the species we used show some variability).

To perform ancestral inference, we used a customized loopy belief propagation algorithm on a factor graph approximation of the model [51]. Parameter estimation was then performed using a generalized EM algorithm. We validated some key results using parsimony analysis (**Fig S10** and data not shown).

For analysis of the resulted model parameters, each context dependent substitution rate was averaged with its reverse complement. For example CAT->CCT is averaged with ATG->AGG. The averaged conditional probabilities are presented in Fig 2, 4, Fig S3 and Fig S4. *A/T gaining* is defined as any of the following substitutions in any flanking contexts: C->A, C->T, G->A, G->T. *A/T loss* in defined as any of the following substitutions in any flanking contexts: A->C, A->G, T->C, T->G. Analysis was generally focused on the *S. cerevisiae* lineage (data on the other lineages are shown in **Fig S3, Fig S5**).

### Evolutionary sequence simulation

In order to estimate the theoretical regional G+C content of *S. cerevisiae* intergenic sequence, we have simulated this sequence using a lineage specific evolutionary probabilistic model learned over the whole intergenic sequence (see above). Specifically, the common ancestor of the *sensu stricto* clade was simulated first based on the learned 2-order markov model. Following this, the sequences of the descendants were simulated based on the simulated ancestor sequence and the corresponding substitution model. Iteratively, the sequences of all species in the phylogeny were simulated, including the extant species. The regional G+C content of the simulated *S. cerevisiae* intergenic sequence is presented in Fig S1.

### Spatial coupling of substitutions

To estimate the coupling between A/T gaining and A/T losing substitutions in the yeast genome, we used our probabilistic model to infer at each genomic position j the posterior probability of each type substitution in the lineage leading to species i from its ancestor (pai):
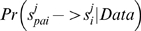



When s^j^_i_ denotes the nucleotide at the j'th genomic position of the i'th species in the phylogeny, and s^j^_pai_ denotes the sequence of the ancestor of this species at the same genomic position.

Given the posterior probabilities we computed for each genomic position j the expected numbers of A/T loss and A/T gain events in the sequence preceding it. This was done using a *horizon* parameter, which was set to 5 bp by default (for alternative horizon values see below):
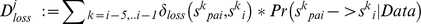


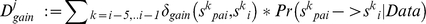



Where the *δ_gain_*, *δ_loss_* functions were given by Table 1, and the *net A/T divergence* of the position was defined as:
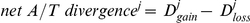



**Table 1:** A/T gain and loss delta parameters.

*s^k^_pai_*	*s^k^_i_*	*δ_loss_*	*δ_gain_*
A/T	C/G	1	0
C/G	A/T	0	1
A/T	A/T	0	0
C/G	C/G	0	0

We then identified all positions with A/T divergence <-0.9 (A/T losing contexts), with A/T divergence >0.9 (A/T gaining contexts) and with conserved A/T content (background). For each such set we computed the probability of A/T gain and A/T loss substitutions using the same inferred posterior probabilities. By using this approach (conditional probability given the events in the preceding 5 bp) we ensured each substitution is counted precisely once. By computing the probabilities for similar events (e.g. A/T gain) given different contexts (A/T losing, A/T gaining, or background), we could robustly asses compensation patterns while controlling for the different basal rates of A/T gain and A/T loss and the general clustering of substitution in the genome.

To statistically assess the coupling between A/T divergence context and A/T losing/gaining substitutions in the *S. cerevisiae* lineage we counted the numbers of A/T gains and A/T losses at A/T gaining and losing contexts:


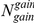
 =  number of A/T gains in A/T gaining contexts


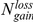
 =  number of A/T losses in A/T gaining contexts


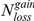
 =  number of A/T gains in A/T losing contexts


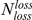
 =  number of A/T losses in A/T losing contexts

In addition we counted the numbers of A/T and C/G occurrences in these contexts:


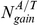
 =  number of A/T's in A/T gaining contexts


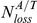
 =  number of A/T's in A/T losing contexts


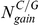
 =  number of C/G's in A/T gaining contexts


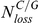
 =  number of C/G's in A/T losing contexts

We wished to test whether the spatial compensation effect is significant even given the general clustering of substitutions. Our null hypothesis was therefore:
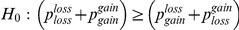



We test it using bootstrapping with 100,000 resamples. At each resample, a set of 
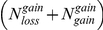
 items are sampled without replacement out of the union of two sets of sizes 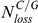 and 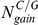 (denoted by A, B respectively). Similarly, we sample without replacement 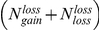 items out of the union of two sets of size 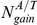, 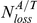
(denoted by C, D respectively). The number of sampled items belonging either to set A or C is collected across all resamples. We end up with 100,000 counts representing the background distribution for the 
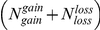
 statistic. P-value for the null hypothesis is calculated by counting the fraction of iterations in which the sampled counts are bigger than 
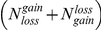
.

Analysis of the robustness of the observed compensation patterns for different values of the horizon parameter is shown in **Fig S11, Fig S12, and Fig S13**.

### Evolutionary theoretical model

To study the hypothesis that selection on dispersed nucleosome encodings drives asymmetric substitution patterns in yeasts, we devised a simple theoretical model. For clarity we describe here the version of the model for low occupancy sequences. For nucleosome DNA the model is the same apart from the fitness function.

First we used a Wright-Fischer dynamics on a population of 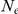 binary sequences of size L, 
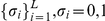
: In each generation there is a probability of 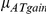 for each site containing 0 to be flipped to 1 and 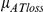 for sites containing 1 to flip to 0. The sequences are then sampled relative to their fitness 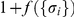, where 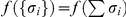 and 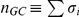 is equivalent to the GC content. We simulated this system for the following parameter set

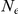
 - (Effective) population size (10000)
- L – Genome size (20)

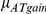
- The rate of G/C -> A/T mutations (3e-7)

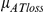
- The rate of A/T -> G/C mutations (7e-7)

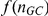
– a fitness function (described below)

We note that the population expected θ parameter may be estimated from the above parameters (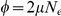 in haploid population, but given the two different mutation rates the empirical theta needs to be corrected). The parameters we used ensured θ<0.04.

The simulation was based on the following procedure:

*Initialize*: Create a population of 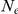 identical sequences of length L. For simplicity sequences use a binary alphabet on A and G. We define the current *reference genome sequence* R using the same initial sequence. We introduce the following counters to accumulate sufficient statistics for computing the rates of A->G and G->A substitutions (N*_A_*, N*_G_* and N_A->G_,N_G->A_, such that the rate will be estimated as N_A->G_ /N*_A_,* N_G->A_ /N*_G_*).

*Sample a new generation*: to create a new generation, we sample 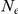 times from the current population using weights that are proportional to the fitness of each individual. For each sampled individual, we introduce mutations with probability 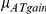 for G loci and 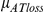
for A loci. Starting after a minimal number of “burn-in” iterations (at least 4 coalescent times) we also incremented N*_A_* and N*_G_* for each sampled individual with the number of A's and G's in the respective sequence.

*Updating the reference genome*: given the new generation population, we tested the frequency of A and G at each of the L genomic loci. Whenever the frequency in the current population is larger than 0.95 and the major allele is different from the reference genome R, we incremented the counter N_A->G_ or N_G->A_ (after the burn-in period) and updated the sequence R.

We end up with counts of A's (N*_A_*), counts of G's (N*_G_*) (in units of generations X loci) and counts of the substitutions between them (N*_A->G_*, N*_G->A_*). Substitution rates are estimated by:
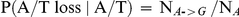


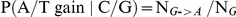



These rates are shown in Fig 5 and Fig S8 for the different fitness landscapes we defined next.

The *goal* landscape is defined symmetrically around an optimal number of G's denoted by n_GC_ and the selection intensity *η* (e.g., X axis in Fig 5B, C, F, G):
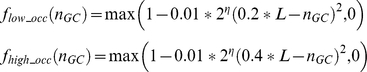



The *threshold* landscape is defined using similar parameters to generate an asymmetric function:
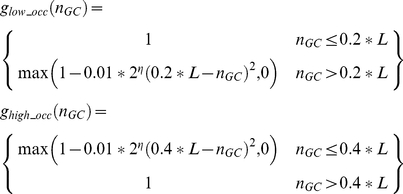



### Analytic approximation

Next, we studied the above model analytically in the regime of low mutation rates. In this regime, drift is the dominating mechanism and we can model the process by assuming the population is represented by a single genome (or GC content). Given the definitions above, the rate at which mutations that increase the GC content enter the population is 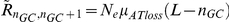



While the rate of mutations that decrease the GC content is
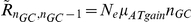



In such drift dominating regime, the fixation probability of a new mutation is:
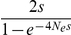
where 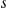 is the marginal fitness of the mutation [52]. Therefore, the rate at which the GC-content increases or decreases is on average
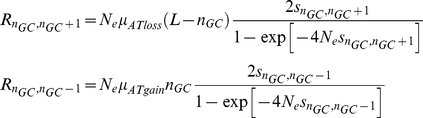



Where 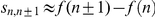.

Thus the set equations for the dynamics of 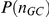 is
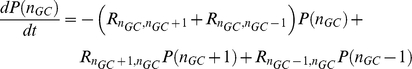



Solving this for the steady state 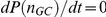 results in
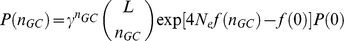



Where 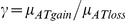 and 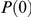 is set by normalization 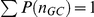. From this distribution of the GC-content one can calculate the average GC-content 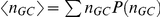 and the substitution rates
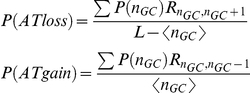



As can be seen in **Fig S8**, the analytical result and Wright-Fischer simulation are in good agreement.

### Allele frequency analysis

We used DNA sequences of 39 *S. cerevisiae* strains sequenced in the Saccharomyces genome resequencing project (SGRP). Here, only intergenic 2 allele SNP's with sequence data from more than 20 strains were considered informative. For each of those SNP's, *major allele* was defined as the most abundant allele in the population. *Minor allele* is defined as the least abundant allele. A/T gaining SNPs were defined when the nucleotide of the major allele was C or G and the minor allele is A or T. A/T losing SNPs were defined reciprocally. All other SNPs were defined as A/T conserving (see illustration in Fig 6). We further subdivided SNPs into two groups: SNP's in G/C flanking context and SNP's with at least one A or T in the flanking contexts, using the reference strain for determining the context. These subgroups are again subdivided to SNP's within low occupancy sequences and SNP's within high occupancy sequences (**Fig 6**). We analyzed the distributions of the frequency of minor alleles of these subgroups separately. In figure 6, shown are the fraction of rare alleles (minor allele frequency <0.20) among A/T gain, A/T loss and A/T conserved SNP's within low or high occupancy sequences. We used a chi-squared test to reject the null of hypothesis that the fraction of rare alleles is the same between A/T gain and A/T loss SNP's.

## Supporting Information

Figure S1 Heterogeneous G+C content. A) Shown is the probability density function of the regional G+C content (20 bp windows) over the intergenic S. cerevisiae sequence (black), over simulated intergenic genomes (red, see methods) and the theoretical binomial distribution (green, p∼0.35, n = 20). B) Shown is the log ratio of the empirical regional G+C content density function and the theoretical density function sampled using the evolutionary sequence simulation. The data suggest that the yeast genome has an excess of both high and low G+C content regions.
(0.32 MB EPS)

Figure S2 Heterogeneous trinucleotides distribution over low and high nucleosome occupancy sequences. A-B) Shown are log ratios of trinucleotide frequencies in low and high occupancy sequences (Y axis) against trinucleotide frequencies in high occupancy sequences (X axis) over TSS proximal sequences (A) and TSS distal sequences (B). Each trinucleotide is depicted by three adjacent color coded squares. Pairs of reverse complimented trinucleotides are averaged and depicted together. In addition to the clear preference of A/T trinucleotides for low occupancy sequences (notice the abundant AAA), we note the differences in G/C trinculeotide preferences between the occupancy groups. (C,D) shown are the log ratios of trinucleotide frequencies (same as A,B) over TSS proximal sequences (C) and TSS distal sequences (D).
(0.39 MB EPS)

Figure S3 Yeast substitution rates are robustly correlated with the flanking nucleotides for all substitution types. Shown are the inferred substitution rates in TSS distal low occupancy sequences for the S. cerevisiae lineage (the gray lineage, x axis), and other sensu stricto lineages (color coded, Y axis), for 16 different flanking nucleotide contexts. The linear fit (dashed line) slopes for each lineage is roughly proportional to its branch length, but the model allows for differences in the substitution rates among lineages. A) A->C, T->G substitutions B) A->G, T->C substitutions C) A->T, T->A substitutions D) C->A, G->T substitutions E) C->G, G->C substitutions F) C->T, G->A substitutions.
(0.90 MB EPS)

Figure S4 A/T gain and loss substitution rates at low and high occupancy loci. Shown are ratios of all substitution rates in low vs. high occupancy loci (Y axis) plotted against the substitution rates at high occupancy loci (X axis) over TSS proximal (A) and distal sequences (B). Each point represents the rate of one substitution (color coded) in loci flanked by the 3′ and 5′ nucleotide depicted above the data point. C,D) Substitution rates by their A/T dynamics in TSS proximal (C) and distal (D) loci. Error bars depict the standard deviation. The trends are identical over transitions and transversions.
(0.66 MB EPS)

Figure S5 A/T gain and loss dynamics in different lineages of the sensu stricto clade. A-F) A/T loss and A/T gain rates over TSS distal (bars) and proximal (gray ticks) for the lineages leading to the following species: S. cerevisiae (A), S. paradoxus (B), S.mikatae (C), S. kudriazevii (D), the common ancestor of S. cerevisiae & S. paradoxus (E), and the common ancestor of S. cerevisiae & S. mikatae (F). G-L) Shown are the average G+C content of the following extant species and inferred ancestors, depicted for 10 levels of S. cerevisiae nucleosome occupancy (Methods): S. cerevisiae (G), S. paradoxus (H), S.mikatae (I), S. kudriazevii (J) the common ancestor of S. cerevisiae & S. paradoxus (K) and the common ancestor of S. cerevisiae & S. mikatae (L).
(0.40 MB EPS)

Figure S6 G/C trinucleotides in TSS proximal low occupancy loci are more likely to be bound by a transcription factor. Shown is the fraction of G/C trinucleotides that are bound by one of the following transcription factors: REB1, UME6, MSN2, MBP1 within TSS distal high occupancy loci (-H), TSS distal low occupancy loci (-L), TSS proximal high occupancy loci (+H), and TSS proximal low occupancy loci (+L).
(0.25 MB EPS)

Figure S7 Coupling of A/T gaining and A/T losing substitutions at TSS-distal sequences. A) Shown is a comparison of the rate of A/T gaining substitutions near inferred sites of A/T losing (black) and A/T gaining (red) substitution, plotted for different ranges of nucleosomes occupancy (X axis). B) Similar analysis of A/T loss substitution rates around inferred A/T gain and A/T loss events.
(0.40 MB EPS)

Figure S8 Theoretical evolutionary model. A-H) Evolutionary simulation in high G+C fitness landscape. Shown are results of a simulation identical to the one described in Figure 5, with the fitness landscape changed to reflect optimality at a G+C content of 40% (higher than the 30% neutral content). I) Theoretical evolutionary model recapitulates the empirical A/T content dynamics observed in the Wright-Fischer simulation. Shown are the substitution rates for each selection intensity of A/T losing mutations (red) and A/T gaining mutations (blue) as approximated analytically (lines), compared to the empirical results (dots).
(0.40 MB EPS)

Figure S9 Allele frequency of A/T gain and A/T loss SNP's differences are robust to rare allele threshold. A–D) Minor allele frequency of non G/C contexts A/T loss, A/T gain and A/T neutral SNP's across low and high occupancy loci. Shown are fraction of minor alleles at low occupancy loci with frequencies smaller than 0.14 (A), fraction of minor alleles at high occupancy loci with frequencies smaller than 0.14 (B), fraction of minor alleles at low occupancy loci with frequencies smaller than 0.3 (C), fraction of minor alleles at high occupancy loci with frequencies smaller than 0.3 (D). E–F) Cumulative distribution function of non G/C, minor allele frequency of A/T loss, A/T gain and A/T neutral SNP's at low occupancy loci (E) and high occupancy loci (F).
(0.37 MB EPS)

Figure S10 Parsimonious inference validates substitution rates heterogeneity and spatial coupling of A/T gain and loss events. A–B) Shown are A/T gain (blue) and A/T loss (red) substitution rates of the S. cerevisiae lineage inferred using parsimony (flanking context independent). Data is shown for TSS distal (A) and proximal (B) DNA sequences of S. cerevisiae, S. paradoxus and S. mikatae. A/T losing rate is ∼50% decreased in low occupancy compared to high occupancy. C–D) Rates of A/T gain and loss events are spatially coupled. Shown is a comparison of the rate of A/T gaining substitution near parsimoniously inferred sites of AT losing (black) and AT gaining (red) substitution, plotted for different ranges of nucleosome occupancy (X axis) across TSS-distal (C) and TSS-proximal (D) loci. This analysis is consistent with the context dependent analysis. E-F) Similar analysis of A/T loss substitution rates around inferred A/T gain and A/T loss events across TSS-distal (E) and TSS-proximal (F) loci.
(0.39 MB EPS)

Figure S11 Spatial coupling between A/T gain and A/T loss (horizon of 1 bp). Shown are the results of an analysis similar to the one shown in Figure 3, but with the horizon used for determining gain/loss context set to only one nucleotide (instead of 5 nucleotides).
(0.42 MB EPS)

Figure S12 Spatial coupling between A/T gain and A/T loss (horizon of 3 bp. Shown are the results of an analysis similar to the one shown in Figure 3, but with the horizon used for determining gain/loss context set to only three nucleotide (instead of 5 nucleotides).
(0.39 MB EPS)

Figure S13 Spatial coupling between A/T gain and A/T loss (horizon of 10 bp). Shown are the results of an analysis similar to the one shown in Figure 3, but with the horizon used for determining gain/loss context set to ten nucleotide (instead of 5 nucleotides).
(0.46 MB EPS)
